# Arsenic and Fluoride Exposure in Drinking Water: Children’s IQ and Growth in Shanyin County, Shanxi Province, China

**DOI:** 10.1289/ehp.9270

**Published:** 2007-01-09

**Authors:** San-Xiang Wang, Zheng-Hui Wang, Xiao-Tian Cheng, Jun Li, Zhi-Ping Sang, Xiang-Dong Zhang, Ling-Ling Han, Xiao-Yan Qiao, Zhao-Ming Wu, Zhi-Quan Wang

**Affiliations:** 1 Shanxi Institute for Prevention and Treatment of Endemic Disease, Linfen, Shanxi Province, People’s Republic of China; 2 Shanyin Center for Disease Control and Prevention, Shanyin, Shanxi Province, People’s Republic of China

**Keywords:** arsenic, children, fluoride, growth, IQ, water

## Abstract

**Background:**

Recently, in a cross-sectional study of 201 children in Araihazar, Bangladesh, exposure to arsenic (As) in drinking water has been shown to lower the scores on tests that measure children’s intellectual function before and after adjustment for sociodemographic features.

**Objectives:**

We investigated the effects of As and fluoride exposure on children’s intelligence and growth.

**Methods:**

We report the results of a study of 720 children between 8 and 12 years of age in rural villages in Shanyin county, Shanxi province, China. The children were exposed to As at concentrations of 142 ± 106 μg/L (medium-As group) and 190 ± 183 μg/L (high-As group) in drinking water compared with the control group that was exposed to low concentrations of As (2 ± 3 μg/L) and low concentrations of fluoride (0.5 ± 0.2 mg/L). A study group of children exposed to high concentrations of fluoride (8.3 ± 1.9 mg/L) but low concentrations of As (3 ± 3 μg/L) was also included because of the common occurrence of elevated concentrations of fluoride in groundwater in our study area. A standardized IQ (intelligence quotient) test was modified for children in rural China and was based on the classic Raven’s test used to determine the effects of these exposures on children’s intelligence. A standardized measurement procedure for weight, height, chest circumference, and lung capacity was used to determine the effects of these exposures on children’s growth.

**Results:**

The mean IQ scores decreased from 105 ± 15 for the control group, to 101 ± 16 for the medium-As group (*p* < 0.05), and to 95 ± 17 for the high-As group (*p* < 0.01). The mean IQ score for the high-fluoride group was 101 ± 16 and significantly different from that of the control group (*p* < 0.05). Children in the control group were taller than those in the high-fluoride group (*p* < 0.05); weighed more than the those in the high-As group (*p* < 0.05); and had higher lung capacity than those in the medium-As group (*p* < 0.05).

**Conclusions:**

Children’s intelligence and growth can be affected by high concentrations of As or fluoride. The IQ scores of the children in the high-As group were the lowest among the four groups we investigated. It is more significant that high concentrations of As affect children’s intelligence. It indicates that arsenic exposure can affect children’s intelligence and growth.

Exposure to arsenic (As) in drinking water has been associated with a decline in intellectual function in children. This association has been established recently on the basis of a cross-sectional study of 201 ten-year-old children in Bangladesh (Wasserman et al. 2004). The authors point out the absence of research on the effects of As on children’s intellectual function. They attribute the lack of data to poorly described dosimetry in reported studies on the neurologic consequences of acute and chronic exposures in adults. Only two other studies, one in Mexico (Calderon et al. 2001) and the other in Taiwan (Tsai et al. 2003), have established tentative adverse associations between As exposure and children’s intellectual function although both studies evaluated only a small number of subjects (*n* < 100). Significant impairments of height, body weight, brain, and intelligence were reported in children poisoned by milk powder containing As in Japan when compared with that of the control group of the same age 16 years after the poisoning event (Wang 1997).

In the 1980s in China, it became known that arsenicosis was occurring in individuals drinking groundwater containing As (Wang and Huang 1994). Shanxi province, where our study was conducted, has been recognized as an area with significant exposure in terms of both As concentration (up to 4,440 μg/L) and population (Sun 2004). Individuals with arsenicosis were first correctly diagnosed by a team of Chinese endemic disease experts during a survey in 1994 in Shanxi. Groundwater As data that accumulated over the years point to two sedimentary basins, Datong (1,350 km^2^) and Jinzhong (800 km^2^), where tube well water drawn from depths between 20 and 50 m often contains As concentrations > 50 μg/L, which is the Chinese drinking water standard (GB 5749-85; Ministry of Health 2007). Groundwater from these basins also contains elevated concentrations of fluoride (up to 10 mg/L) but not always together with elevated concentrations of As. The population residing in Datong and Jinzhong basins is 932,086, although only 60% of the total population (569,685) was exposed to high-As concentrations in groundwater because of the heterogeneity of groundwater As distribution. To date, 3,998 individuals with arsenicosis, including children, have been identified, with most living in rural areas.

Here, we report the results of an ecologic study on the intelligence and growth in 720 children between 8 and 12 years of age from Shanxi province, China. Subjects were drawn from control (*n* = 196), high-fluoride (*n* = 253), medium-As (*n* = 91), and high-As (*n* = 180) groups. Our study expands the existing but very limited literature base on the effect of As on the intellectual function of children, and reports a measurable reduction of IQ (intelligence quotient) scores due to As exposure. We initially included fluoride in our investigation because of the high concentrations of fluoride contained in some of the groundwater wells in our study area. To the best of our knowledge, there is no literature published in English that shows fluoride exposure has an effect on the intellectual function of children, although literature published in Chinese points to significant impairment of children’s intellectual function (Li et al. 2004; Liu et al. 2000; Yu et al. 1998), and growth (Qian et al. 1989; Xu and Huo 2000), based on studies of children with fluorosis.

## Methods

### Overview

Our current project is part of a larger ongoing evaluation of the health effects of an As study supported by the Shanxi Natural Science Foundation. The study was approved by our institute’s Institutional Review Committee. As in most rural areas in Shanxi in central northern China, people in villages live in brick–concrete houses with floors made of brick and roofs made of tiles. Members of extended families live in clusters of individual houses surrounded by family farmland. Each extended household has one or more tube wells. This region is very poor even by Chinese standards, with an annual income of approximately US$120 per family. Before conducting this study, we obtained informed written consent from parents and children by arranging meetings through local health clinics, village leaders, and teachers from children’s school.

### Subjects

In 2003, 524 children between 8 and 12 years of age were recruited from Gucheng township in Shanyin county to form the study groups comprising individuals who had been exposed to either As or fluoride. There are 23 villages in Gucheng, with a population of 17,321 and a land area of 220.9 km^2^. Previous survey data on water containing As and fluoride were used to identify 9 villages as target areas for recruitment. The average content of As and fluoride in drinking water in the village was used as the basis to form the three study groups: high-As group from Dongxingzhuan (DXZ), Nanwanzhuang (NWZ), and Silizhuang (SLZ) villages; medium-As group from Yangjuantou (YJT), Yangjuanpu (YJP), and Hongqitun (HQT) villages; and high-fluoride group from Housheduo (HSD), Nanjufang (NJF), and Yuanying (YY) villages (Table 1). Approximately 80% of children in the 8- to 12-year-old age group in the 9 villages agreed to participate in this study (Table 1). With this large number of recruited individuals, we hope to avoid sampling bias. Children from the control group (*n* = 196) were enrolled from 3 villages in nearby Heshengbao township (~ 13.8 km from Gucheng) with a population of 11,601 and a land area of 169.3 km^2^, also in Shanyin county. Approximately 75% of the children between 8 and 12 years of age from these villages in Heshengbao were recruited. We chose Heshengbao as the control group because the data on As and fluoride in water showed uniformly low As and fluoride concentrations (Table 1; Wang et al. 2003). In addition, all groups lived in rural areas with similar geographic and cultural conditions and a comparable level of socioeconomic development (Table 1). All children were currently attending school.

### Procedure

Children participated in the assessments described below and received medical examinations by a team of trained personnel with medical backgrounds. Each team member was assigned to a single task that included administering the IQ test, measuring height, weight, chest circumference, and lung capacity. In addition, all children agreed to provide spot urine samples for the measurement of urinary As and urinary fluoride. Urine samples were stored and transported on ice to the laboratory. Information on family demographics (e.g., parental age, education, occupation, housing type) was obtained from interviews of parents during enrollment of their children in the study (Table 1). Information on income was based on 2000 census data (Table 1; Statistical Information of Shanxi 2007). Unfortunately, we did not include questions on other sources of drinking water for each child other than on the household well.

### Measures

#### Water analyses

We collected water samples from 10–30% of wells randomly selected from our study area during a groundwater sampling study that was shown to provide a statistically representative mean for each village (Sun et al. 2003). A shortcoming of this study design was that we could not evaluate and individual child’s level of exposure. This prevented us from establishing a dose–response relationship. Briefly, samples were collected in 50-mL plastic bottles and 1-mL of 7 N high-purity HCl was added for preservation. Water containing As was analyzed by hydride generation atomic fluorescence spectrometry (HG-AFS) on an AFS-820 (Jitian Instrument Company, Beijing, China) with a limit of detection (LOD) of 0.06 μg/L ± 2.4%. Water containing fluoride was analyzed by a fluoride ion selective electrode with an LOD of 50 μg/L ± 2%.

#### Urinary measurements

We determined As content in urine using HG-AFS, according to a standard protocol (Cheng et al. 2004), with an LOD of 0.06 μg/L ± 4%. The fluoride content in urine was also determined using a fluoride ion selective electrode with an LOD of 50 μg/L ± 2%.

#### Children’s IQ scores

The scores from children’s intelligence tests were measured by the Combined Raven’s Test - The Rural in China (CRT-RC_2_) method. This method is based on the Raven’s Standard Progressive Matrices (SPM) and Color Progressive Matrices (CPM) (Raven et al. 1983) for fluid intelligence and was widely adopted in China after modifications were made for cultural, ethnic, and language differences (Chen 2002). Briefly, 72 questions in six groups of the CRT-RC_2_ corresponded to the CPM groups A, A_B_, and B, and the SPM groups C, D, and E in the original Raven matrices. A rural version of the test was used for this study because it was the standard test for children between 4 and 15 years of age who lived in rural settings in China. This version has also been widely used for IQ tests for children in China with hearing and speech disabilities and mental retardation. Children’s IQ scores were calculated from the raw scores of the CRT-RC_2_ tests by applying the 1997 version of a common model established for Chinese children. The common model is essentially a standardized scale for Chinese children established using the same methodology as the Wechsler Intelligence Scale (Wechsler 1991). The children’s IQ scores were divided according to the scale into the following categories: low intelligence, ≤ 69; marginal intelligence, 70–79; below medium, 80–89; medium, 90–109; above medium, 110–119; good, 120–129; excellent, ≥ 130. We recognize that this scale is for nonverbal reasoning and is only measures certain aspects of intelligence. However, no other standardized IQ tests or common models have been developed for rural Chinese children.

#### Children’s growth

Children’s growth function was grouped according to five levels (He 1994) by applying a Z-score method (Z = ±1, Z = ±2) using a standardized procedure for Chinese children (Ye 2000). Briefly, dimensionless Z-scores were calculated on the basis of measurement values using the following equation:


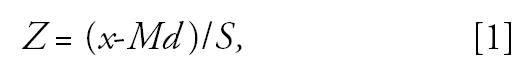


where *x* is the individual child’s growth measure (i.e., height, weight), *Md* is the medium, and *S* the standard deviation. Therefore, positive Z-scores represent above-median values and negative Z-scores represent below-median values. The Z-score transformation above allowed us to convert the measurement data into rank data. We ranked the children’s growth functions according to the following five categories: lower growth, ≤ 2; lower-medial growth, –2 to –1; medial growth, –1 to 1; upper-medial growth, 1 to 2; and upper growth, ≥ 2. Statistical analysis was then performed on the rank data.

### Statistical analyses

Data were analyzed by SPSS for Windows 10.0 (SPSS, Chicago, IL, USA) and CS2000 (Department of Public Health, Shandong University, Shandong, China) by the Statistics Teaching and Research Section of Public Health College of Shandong University. One-way analysis of variance (ANOVA) was performed on the log-transformed data; the Kruskal-Wallis test was also used. Regression ANOVA was performed on the log-transformed data. Paired *q*-test, chi-square test, and *t*-test were used to compare differences between any of the two study groups. Coefficients were calculated using the Spearman rank-order correlation test. All values were transformed back to the arithmetic scale for reporting purposes. We did not perform a multiregression analysis.

## Results

### Sample characteristics

In Table 1 we present descriptive information on demographics, income, parents’ education, and the variables for As, fluoride, and manganese in water. The average age of children was 10 years, and approximately half the samples were male. Children on average have been exposed since birth to the well being used by the household. On average, parents reported having 6 years of primary school education, with an average income from farming of approximately US$150 per year (1,200 Yuan RMB per year). The average household consisted of 3.6 persons residing in a brick–concrete dwelling.

### Exposure characteristics

The mean water As concentrations were 2, 3, 142, and 190 μg/L in the control, high-fluoride, medium-As, and high-As groups, respectively (Table 2). The mean urinary As concentrations were 10, 6, 46, and 73 μg/L in the control, high-fluoride, medium-As, and high-As groups, respectively. The mean water fluoride concentrations were 0.5, 1.7, and 0.9 mg/L in the control, medium-As, and high-As groups, respectively, but was 8.3 mg/L for the high-fluoride group (Table 2). The mean urinary fluoride concentrations were 1.5, 2.8, and 1.0 mg/L in the control, medium-As, and high-As groups, respectively, but was 5.1 mg/L in the high-fluoride group. Thus, children in the medium-As group were exposed to some amount of fluoride and showed elevated urinary fluoride concentrations. Fewer water samples than urine samples were analyzed for As and fluoride. Both water and urine were randomly selected for sampling in order to determine the mean values of the exposure group. An unfortunate shortcoming is that the nature of this study design prevented us from establishing a dose–response relationship.

### The distribution of children’s IQ scores

**Figure 1:**
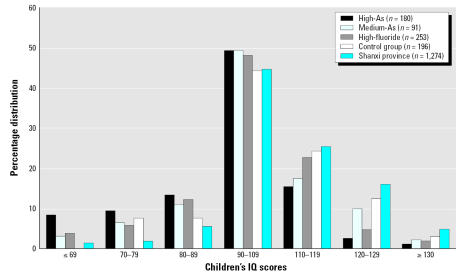
Frequency distribution of IQ in 10-year-old children from Shanyi county, Shanxi, China, who were exposed to high-As water, medium-As water, and high fluoride compared with control group with low-As and low-fluoride, as well as children residing in Shanxi province, China.

In each study group, the number of children who were administered IQ tests was greater than the number of analyses performed on water and urinary As and fluoride that represented the mean values of exposure (Tables 2, 3). The distribution of children’s IQ scores is slightly skewed in the control group: 15% (*n* = 30) with IQ score < 90; 44% (*n* = 87) with IQ score between 90 and 109; and 41% with IQ score > 109 (Table 3; Figure 1). The percentage of children with IQ score > 109 decreased to 30, 30, and 19% in the high-fluoride group, the medium-As group, and the high-As group, respectively. A chi-square test (significance level, α = 0.0083) shows that this disturbingly low percentage of children with IQ score > 109 in the high-As group is significantly lower than that in the high-fluoride group (χ^2^ = 5.77, *p* < 0.05) and that in the control group (χ^2^ = 19.33, *p* < 0.01) but not significantly different from that of the medium-As group. The percentage of children with IQ score < 70, or children with intellectual disabilities, increased significantly from 0% in the control group to 4, 3.3, and 8.3% in the high-fluoride, medium-As, and high-As groups, respectively. Based on a sampling of 1,274 children in Shanxi, the percentage of children with IQ score < 70 is 1.3% (Chinese Center for Disease Control and Prevention 2006).

In the four groups, the change in the distribution of children’s IQ scores is also reflected in the mean values of IQ scores in decreasing order from 105 ± 15 (*n* = 196) in the control group to 101 ± 16 (*n* = 253) in the high-fluoride group and 101 ± 16 (*n* = 91) in the medium-As group, and finally to 95 ± 17 (*n* = 180) in the high-As group. The 10-point reduction in IQ scores in the high-As group with a 190-μg/L mean exposure to water As (Table 1) compared with the control group is significant (*q* = 8.42, *p* < 0.01). The 4-point reduction in IQ scores in both the high-fluoride group (*q* = 4.99, *p* < 0.01) and the medium-As group (*q* = 2.94, *p* < 0.05) compared with the control group is also significant. The difference in IQ scores between any of the two groups except that between the high-fluoride group and the medium-As group is significant by the *q*-test (*p* < 0.05 or 0.01).

### The distribution of children’s growth

The distribution of the four parameters that indicate children’s growth and development generally showed that exposure to As and fluoride in water had a negative impact, although the effect is neither as striking as the impairment of IQ nor always statistically significant (Table 4). Chest measurements showed the least difference or no statistically significant differences in average rank values among the four groups (Table 5). Average weight, height and lung capacities in children exposed to either As or fluoride, or both, however, are always lower than the average rank values for children in the control group (Table 5). The statistically significant differences were found in the following comparisons: Children’s height in the control group was significantly higher than that in high-fluoride group (*p* < 0.05); children’s weight in the control group was significantly higher than that in the high-As group (*p* < 0.05); children’s lung capacity in the control group was significantly higher than that in the medium-As group (*p* < 0.05).

### Relationship between As and fluoride exposure and intellectual function

Urinary As concentrations correlated positively with water As concentrations in children in all study groups. Similarly, urinary fluoride concentrations also correlated positively with water fluoride concentrations in children in all study groups. The Spearman correlation coefficient that was calculated between urinary As concentrations and children’s IQ in the control and high-As groups was –0.201 (*p* < 0.01) and was unadjusted for other factors that may contribute to IQ. This negative correlation suggests that As exposure via water lowers IQ score. Similarly, a Spearman correlation coefficient of –0.107 (*p* < 0.05) was found between urinary fluoride and IQ scores in children in the control and the high-fluoride groups. This negative correlation suggests that fluoride exposure also lowers IQ. These negative correlations between IQ and urinary As and between IQ and urinary fluoride indicate that exposure to high levels of As or fluoride, or both, could affect children’s intelligence. No statistically significant negative correlations were found between IQ and urinary As or between IQ and urinary fluoride in children in the control and medium-As groups. This, in part, is because of the small sample size of the medium-As group comprising only 91 children (Table 3).

## Discussion

This study indicates that exposure to fluoride in drinking water is associated with neurotoxic effects in children. A literature search on fluoride and intelligence in the PubMed database (http://www.ncbi.nlm.nih.gov/pubmed/) returned studies conducted only in China and none from western literatures. Animal experiments showed that fluoride is neurotoxic to rats and damages brain functions (Cheng et al. 2002). A survey of newborn infants from Zhao county in Heilongjiang province in northeastern China, an area with a high rate of fluorocosis, found that exposure of mothers to high concentrations of fluoride affected neurobehavioral development and agonistic muscle tension development in infants. Agonistic muscle tension is often affected when brain damage occurs, which is consistent with the finding that, in China, exposure to fluoride affects children’s intelligence (Liu et al. 2000; Yu et al. 1998). In our follow-up study of this population, we are working toward establishing a dose–response relationship between exposure to fluoride in water and children’s IQ.

It is less surprising that exposure to fluoride affected children’s growth function, especially height. Previous studies have demonstrated multiple effects of exposure to high concentrations of fluoride on children’s morphology, growth and development, and on bones and teeth (Qian et al. 1989; Xu and Huo 2000). This is because fluoride accumulates in bone and reduces calcium uptake, thereby influencing growth.

When Ma et al. (1994) exposed adult rats to As, the authors found that baby rats developed abnormal nerve construction in the brain cortex and were slower to gain weight compared with normal rats. Although the results of the animal study of Ma et al. suggest the possibility that brain damage in children could be caused by maternal exposure to As, there is no evidence to date to support this hypothesis. Li and An (2005) found that As can pass through the blood–brain barrier and the placenta barrier, thereby, potentially affecting fetus brain development.

Because of the decline we found in the intellectual function of children from exposure to As, it appears that this exposure is more significant than fluoride exposure. We note that urinary flouride in the medium-As group displayed a geometric mean of 2.8 mg/L and is approximately twice that of the control group, with a geometric mean of 1.5 mg/L (Table 2). This somewhat elevated level of fluoride exposure did not appear to greatly affect the IQ of children in this group, because the decline in IQ scores from 105 to 101 to 95 was observed in the control group. The mean values for water As in the three groups are as follows: control group, 2 μg/L; medium-As group, 142 μg/L; and high-As group, 190 μg/L (Table 2). In other words, the medium-As group would have even lower IQ scores if exposure to higher concentrations led to severe impairment of intelligence, but it did not. Taken together, our results highlight the significant impact of As exposure on children’s intelligence. Even in water that may contain other agents such as fluoride that also impair intelligence, As remains the primary factor in affecting children’s intelligence.

### Limitations

Children’s intelligence, growth, and development can be influenced by many factors such as inheritance, nutrition, geography, education, and society. Our current study sampled a homogenous rural population in Shanxi, and therefore, the influence of socioeconomic and genetic factors was expected to be minimal, but we cannot completely exclude the influence of such factors. After adjustment for these factors, Wasserman et al. (2004) found that exposure of a Bangladeshi 10-year-old child to only 5 μg/L of drinking-water As led to a reduction in intellectual function. A report from Thailand pointed out that the intellectual function of children correlated with As exposure, with about 14% of variance resulting from As exposure after removing confounding factors (Siripitayakunkit 1999). Additionally, we recognize that children in our study groups attend school and therefore are exposed to different levels of As while not at home. All the complications and limitations of our study design, however, would not lead to systematic errors that would challenge the main findings: that 10-year-old Chinese children’s IQ scores were lowered by 5–10 points when they were exposed to drinking water containing 150–200 μg/L As. However, we emphasize the need for more careful evaluation of the effects of fluoride on intelligence. Even though urinary As was low (Table 2) in 101 of 253 children in the high-fluoride group, we cannot exclude the possibility that the urinary As could be higher in the 142 children who were not evaluated.

## Figures and Tables

**Table 1:** Study parameters for villages in Gucheng township.^a^

	High-As group b			Medium-As group^b^			High-fluoride group^b^				
Village	DXZ	NWZ	SLZ	YJT	YJP	HQT	HSD	NJF	YY	Control group	*p*-Value
No. of males	20	12	5	31	68	36	14	46	32	112	0.07^c^
No. of females	26	12	16	27	59	32	23	42	23	84	
Sum^d^	46 (58)	24 (30)	21 (26)	58 (74)	127 (156)	68 (85)	37 (46)	88 (112)	55 (69)	196 (257)	
Average age (years)	10.0 ± 1.3	10.0 ± 1.2	9.8 ± 1.2	10.0 ± 1.2	10.1 ± 1.3	10.0 ± 1.5	10.1 ± 1.4	9.9 ± 1.6	10.1 ± 1.5	9.9 ± 1.5	0.65^c^
Average income (RMB/year)	1,179 ± 223	1,240 ± 187	1,194 ± 235	1,150 ± 205	1,238 ± 241	1,164 ± 216	1,239 ± 252	1,156 ± 217	1,209 ± 177	1,240 ± 215	0.06^c^
Parents’ education (years)	6.3 ± 2.7	6.4 ± 2.3	6.6 ± 2.5	6.8 ± 2.5	6.5 ± 2.5	6.6 ± 2.3	6.8 ± 2.9	6.8 ± 2.5	6.6 ± 2.8	6.8 ± 2.6	0.15^c^
Water As (μg/L)	166 ± 160	250 ± 230	176 ± 101	157 ± 120	141 ± 111	129 ± 103	3 ± 1	3 ± 2	5 ± 4	2 ± 3	
Water fluoride (mg/L)	1.0 ± 0.6	0.9 ± 0.5	0.9 ± 0.3	2.3 ± 1.5	1.3 ± 0.7	1.6 ± 1.1	7.4 ± 2.1	8.6 ± 0.9	9.2 ± 1.9	0.5 ± 0.2	
Exposure time (years)	10.0 ± 1.3	10.0 ± 1.2	9.8 ± 1.2	10.0 ± 1.2	10.1 ± 1.3	10.0 ± 1.5	10.1 ± 1.4	9.9 ± 1.6	10.1 ± 1.5		

Abbreviations: DXZ, Dongxingzhuan; HSD, Housheduo; NWZ, Nanwanzhuang; SLZ, Silizhuang; YJT, Yangjuantou; YJP, Yangjuanpu; HQT, Hongqitun; NJF, Nanjufang; YY, Yuanying.

a Values are reported as mean ± SD for all parameters unless otherwise noted.

b Mean water manganese concentration across all villages was 43 ± 36.5 μg/L.

c Study groups vs. control groups, *p* > 0.05; there is no statistical difference for age, income, or parents’ education.

d The total number of children between 8 and 12 years of age in each village is listed in parentheses.

**Table 2:** Arsenic (μg/L)and fluoride (mg/L) concentrations in drinking water and urine from subjects in Shanxi province, China.

Group	Water As [*n* (mean ± SD)]	Water fluoride [*n* (mean ± SD)]	Urinary As [*n* (GM ± SD) ]	Urinary fluoride [*n* (GM ± SD)]
High-As	50 (190 ± 183)** [14, 502]	50 (0.9 ± 0.5) [0.3, 1.8]	86 (73 ± 3)* [17, 595]	73 (1.0 ± 1.7) [0.2, 3.6]
Medium-As	30 (142 ± 106)* [7, 303)	30 (1.7 ± 1.1)** [0.5, 3.8]	50 (46 ± 3)* [9, 315]	50 (2.8 ± 1.9)* [0.4, 6.6]
High-fluoride	21 (3 ± 3) [1, 10]	21 (8.3 ± 1.9)** [3.8, 11.5]	101 (6 ± 2) [2, 20]	106 (5.1 ± 2.0)* [1.6, 11.0]
Control	11 (2 ± 3) [1, 10]	11 (0.5 ± 0.2) [0.2, 1.1)	120 (10 ± 2) [3, 47]	110 (1.5 ± 1.6) [0.4, 3.9]

GM, geometric mean.

Numbers in brackets are lowest and highest observed values. Significant difference from control group:

* *p* < 0.05,

** *p* < 0.01.

**Table 3:** Distribution of children 8 –12 years of age based on intellectual function.

				Distribution (%)						
Group	*n*^a^	Age in years (mean ± SD)	IQ (mean ± SD)	≤ 69	70–79	80–89	90–109	110–119	120–129	≥ 130
High-As	180	9.9 ± 1.5	95.1 ± 16.6*	8.3	9.4	13.3	49.4	15.6	2.8	1.1
Medium-As	91	9.8 ± 1.4	100.6 ± 15.6**	3.3	6.6	11.0	49.5	17.6	9.9	2.2
High-fluoride	253	9.9 ± 1.4	100.5 ± 15.8**	4.0	5.9	12.3	48.2	22.9	4.7	2.0
Control	196	9.9 ± 1.5	104.8 ± 14.7	0	7.7	7.7	44.4	24.5	12.8	3.1
Shanxi	1,274	9.0 ± 0.82	108.0 ± 14.0^b^	1.3	2.0	5.7	44.8	25.4	16.0	4.7
China	37,288	9.0 ± 0.94	103.5 ± 17.7^c^	3.6	5.4	10.5	43.1	19.8	12.6	5.0

a Number of children tested.

b The average IQ of children in Shanxi was determined to be 108.0 ± 14.0 in 2005, which is statistically different from that of the control group (*u* = 3.2, *p* < 0.01).

c The average IQ of Chinese children was determined to be 103.5 ± 17.7 in 2005, which is not statistically different from that of the control group (*u* = 0.996, *p* > 0.05).

Significant difference from control group:

* *p* < 0.01,

** *p* < 0.05.

**Table 4:** Rank distribution of children 8–12 years of age based on growth and development factors.

		Distribution (%)^a^				
Indicator, group	*n*	Lower	Lower medial	Medial	Upper medial	Upper
Height						
Medium-As	107	7.5	18.7	70.1	3.7	0
High-fluoride*	278	7.2	28.4	60.1	2.9	1.4
High-As	190	7.9	21.6	67.4	3.2	0
Control*	205	5.4	20.0	65.9	5.9	2.9
Weight						
Medium-As	107	0.9	15.9	75.7	3.7	3.7
High-fluoride	278	0.4	14.0	77.0	5.8	2.9
High-As*	190	0	18.4	78.9	2.6	0
Control*	205	1.0	5.4	81.5	6.3	5.9
Chest circumference						
Medium-As	106	0	10.4	82.1	4.7	2.8
High-fluoride	278	0.7	13.3	79.1	5.4	1.4
High-As	190	0	11.6	82.1	5.8	0.5
Control	205	1.0	16.1	75.1	4.9	2.9
Lung capacity						
Medium-As*	89	0	7.9	88.8	2.2	1.1
High-fluoride	277	0	5.1	78.7	13.0	3.2
High-As	108	0	3.7	86.1	8.3	1.9
Control*	205	1.5	7.8	64.9	19.0	6.8

*n* = number of children tested.

a Values are based on percent of median value for each indicator.

* Significant difference, *p* < 0.05.

**Table 5:** Average rank of growth and development indicators.

	Height		Weight		Chest measurement		Lung capacity	
Groups	*n*	Average rank	*n*	Average rank	*n*	Average rank	*n*	Average rank
Medium-As	107	399	107	377	106	404	89	294*
High-fluoride	278	369*	278	389	278	387	277	343
High-As	190	386	190	355*	190	395	108	327
Control	205	418*	205	430*	205	380	205	362*

*n* = number of children tested.

* Significant difference, *p* < 0.05.
